# Diabetes self-management: a qualitative study on challenges and solutions from the perspective of South African patients and health care providers

**DOI:** 10.1080/16549716.2022.2090098

**Published:** 2022-07-20

**Authors:** Tiny Masupe, Sunday Onagbiye, Thandi Puoane, Absetz Pilvikki, Helle Mölsted Alvesson, Peter Delobelle

**Affiliations:** a Department of Family Medicine & Public Health, Faculty of Medicine, University of Botswana, Gaborone, Botswana; b School of Public Health, University of the Western Cape, Bellville, South Africa; c The Africa Unit for Transdisciplinary Health Research (AUTHeR), North-West University, Potchefstroom Campus, Potchefstroom, South Africa; d Collaborative Care Systems Finland, Helsinki, Finland; e Department of Global Public Health, Karolinska Institutet, Stockholm, Sweden; f Chronic Disease Initiative for Africa, University of Cape Town, Cape Town, South Africa; g Department of Public Health, Vrije Universiteit Brussel, Brussels, Belgium

**Keywords:** Self-management, Type-2-diabetes, barriers, patient-derived solutions, multidisciplinary, chronic disease care

## Abstract

**Background:**

Health education and self-management are among key strategies for managing diabetes and hypertension to reduce morbidity and mortality. Inappropriate self-management support can potentially worsen chronic diseases outcomes if relevant barriers are not identified and self-management solutions are not contextualised. Few studies deliberately solicit suggestions for enhancing self-management from patients and their providers.

**Objective:**

This qualitative study aimed to unravel experiences, identify self-management barriers, and solicit solutions for enhancing self-management from patients and their healthcare providers.

**Methods:**

Eight in-depth interviews were conducted with healthcare providers. These were followed by four focus group discussions among patients with type-2- diabetes and or hypertension receiving chronic disease care from two health facilities in a peri-urban township in Cape Town, South Africa. The Self-Management framework described by Lorig and Holman, based on work done by Corbin and Strauss was used to analyse the data.

**Results:**

Patients experienced challenges across all three self-management tasks of behavioural/medical management, role management, and emotional management. Main challenges included poor patient self-control towards lifestyle modification, sub-optimal patient-provider and family partnerships, and post-diagnosis grief-reactions by patients. Barriers experienced were stigma, socio-economic and cultural influences, provider-patient communication gaps, disconnect between facility-based services and patients’ lived experiences, and inadequate community care services. Patients suggested empowering community-based solutions to strengthen their disease self-management, including dedicated multidisciplinary diabetes services, counselling services; strengthened family support; patient buddies; patient-led community projects, and advocacy. Providers suggested contextualised communication using audio-visual technologies and patient-centred provider consultations.

**Conclusions:**

Community-based dedicated multidisciplinary chronic disease healthcare teams, chronic disease counselling services, patient-driven projects and advocacy are needed to improve patient self-management.

## Background

Type 2 diabetes (T2D) and hypertension (HTN) lead to morbidity and mortality due to their complications [1] and higher health services utilization [2]. The World Health Organization (WHO) estimates that diabetes was directly responsible for 1.5 million deaths globally in 2019 [3]. The highest contributors to global mortality and disability-adjusted life years (DALYs) due to diabetes included increased Body Mass Index (BMI) and behavioural risk factors, with sub-Saharan Africa recording the second-highest age-standardized mortality [4]. The Burden of Disease Research Unit at the South African Medical Research Council also reported increased mortality from diabetes between 1997 and 2010 due to population growth and change in age structure [5]. Diabetes is estimated to affect 10.1% of South Africans aged 15 years and above [6].

Health education and self-management (SM) are among key strategies for T2D and HTN management according to the WHO [7], aimed at reducing disease complications. Self-management however, is considered poor in Sub-Saharan Africa [8] and challenging in South Africa [9]. Physiological benefits to patients in SM programmes are documented but harm can occur if SM is not patient-specific [10]. With the advent of Covid-19, the importance of self-management has become even more pronounced, especially among diabetes patients. A study in India showed that the usual patient-centred care for diabetes patients needed to adapt to more self-management [11] techniques at home where patients have to apply the self-management principles, showing that self-management remains critical even for infectious diseases.

Self-management is described based on tasks that fall within three categories: medical/behavioural management, role management, and emotional management [12]. The medical/behavioural role entails proper use of medication for disease control and adopting positive health behaviours geared towards slowing down disease progression. Role management defines new long-term partnerships between patients and healthcare professionals where the patient plays a role of a partner, accurately and truthfully reporting any changes in symptoms. The emotional role consists of the patient’s emotional reactions to chronic disease.

While self-management support is effective in improving clinical outcomes, it can potentially worsen chronic disease outcomes when barriers are not identified and self-management solutions are not population-specific [13]. Few studies however deliberately solicit suggestions for optimizing self-management from patients and their providers [14]. This qualitative study aimed to unravel experiences, identify self-management barriers, and solicit solutions for enhancing self-management of T2D/HTN from patients and their healthcare providers.

## Methods

### Study setting

This study was part of the formative phase of a larger study (A people-centred approach to Self-Management And Reciprocal Learning for the prevention and management of Type 2 Diabetes – SMART2D) which aimed to strengthen capacity for T2D care and self-management among people at risk of or diagnosed with T2D in three different income settings in Uganda, Sweden and South Africa [15]. The study was conducted in two community health centres providing chronic disease care for T2D/HTN in a predominantly black African township (99.5%) in Cape Town, South Africa. The township had an estimated population of 400,000 in 2011 (nearly 96.5% speaking isiXhosa) with high rates of unemployment (est. 35.7% in 2017), 19% having no income and over half of the 118,000 households living in informal dwellings [16], 23% living in dwellings without piped water, and around 62% of residents being rural to urban migrants [17], mostly originating from the Eastern Cape. The township has seven government-run public healthcare facilities, with previous studies indicating a high prevalence of HTN (88.6%) and T2D (12.5%) among adults aged 18 and above [17,18]. The health system is typically low-resourced with nurses being responsible for day-to-day management of these patients and doctors seeing mainly difficult cases. Following initial diagnosis and treatment, non-communicable disease (NCD) patients with a stable disease typically receive care from nurses in chronic disease clubs at facility level. All health services are free at the point of access, including medications for diabetes and hypertension. The role of the private sector in this setting is negligible if any, but there are non-governmental organizations (NGOs) which run some chronic disease services, including collection and delivery of medications.

In 2013, the National Department of Health embarked on a primary healthcare re-engineering process in line with the call for Universal Health Coverage, which aimed, among others, to strengthen the management and control of chronic diseases at primary health care level. The chronic disease clubs came about as part of implementation of this process [18], with the aim of empowering patients with diabetes with skills and knowledge to manage their diabetes. The model was expanded to include other chronic diseases such as hypertension, asthma, and epilepsy. In the clubs, patients with chronic diseases are monitored for risk factor management, anthropometric measurement, biomarkers such as glucose and cholesterol, and health education for SM. The clubs are free to attend and operate on a monthly basis.

### Study design and population

The study involved conducting eight in-depth interviews (IDIs) with healthcare workers (HCWs) and four mixed focus group discussions (FGDs) with male and female patients diagnosed with T2D/HTN attending the two study facilities. In our study, we used the SM framework by Lorig and Holman to situate our study and analyse the findings. We ended the study with a recommendation for a contextualised framework for SM by looking at other self-management theories. Creswell [19] alludes to the inductive approach, whereby information is collected from participants using interviews or field notes, data is analysed to form themes or categories, broad patterns or generalizations are identified, and finally compared with the literature. The essence of the patients’ experience with SM starting from the day of diagnosis; the emotional reactions to the diagnosis; and subsequent experiences when engaging with the healthcare providers and health system in the chronic disease clubs which led participants to identify potential solutions to address challenges with their self-management. In this paper, we focus on their suggestions for improving self-management, based on their lived experience.

Participants were selected purposively to ensure the provision of rich information on self-management for T2D/HTN and care provision. Selection of patients for the FGDs used purposive sampling for maximum variation, with inclusion criteria being over 18 years, diagnosed with T2DM/HTN for at least six months to ensure self-management experience, and using healthcare services in the selected facility for more than six months prior to enrolment. Patients without diabetes and or hypertension, patients with type 1 were excluded. Variability within the group was enhanced by encouraging participation of younger (<40 years) and older (>60 years) patients. Attempts were made to include both males and females; patients with both well controlled diabetes (HbA1c <7%) and poorly controlled diabetes (HbA1c >8%); and patients with well-controlled blood pressure of <130/90 mmHg and poorly controlled blood pressure (>130/90 mmHg). This information was obtained from the HCWs who assisted with patient recruitment during T2DM/HTN ‘club’ days (when patients attended the clubs for their monthly reviews). The sister in charge of the clinic and the health promoter were informed of the study and its objectives. The sister then addressed the patients waiting for consultation and briefed them on the study.

The study population for IDIs consisted of all HCWs caring for T2D/HTN patients at the facilities, except doctors who were unavailable for interviews. All healthcare providers interviewed were over the age of 18, with the youngest being the dietician, followed by the health promoter, and the oldest being a clinical nurse practitioner 1. Three clinical nurse practitioners (CPNs), one dietician, and one health promoter were interviewed in Facility A. In Facility B, three interviews were conducted with two CPNs and one social worker. The staff complement was slightly different among both sites. The dietician in Facility A also serviced Facility B, and the health promoter in Facility B did not consent to be interviewed (Table 1).

**Table 1.** Characteristics of HCWs in the in-depth interviews.

Facility name	Cadre	Gender	Years of Workexperience
Facility A	Health promoter	Male	8
Facility A	Dietician	Female	2
Facility A	Clinical nurse practitioner 1	Female	>20
Facility A	Clinical nurse practitioner 2	Female	>20
Facility A	Clinical nurse practitioner 3	Female	10
Facility B	Facility manager/ clinical nurse practitioner	Female	>20
Facility B	Clinical nurse practitioner 4	Female	>20
Facility B	Social worker	Female	11

For patient FGDs, purposive sampling was used with inclusion criteria being over 18 years of age, at least six months of T2D/HTN diagnosis, and using healthcare services in the selected facility before enrolment. Four FGDs were conducted (two per facility) with 43 participants, mainly including females (only eight males). The patient age range was 38–75 years, and patients had been diagnosed with T2DM/HTN between six months and 21 years prior to data collection (Table 2).

**Table 2.** Composition of the FGDs.

Group number	Number of participantsper group	Facility	Gender	
			Female	Male
1	13	Facility B	9	4
2	10	Facility B	10	0
3	8	Facility A	6	2
4	12	Facility A	10	2

HCWs at each facility assisted with patient recruitment during monthly review visits, and a quiet room was used for consenting patients meeting the inclusion criteria before conducting the FGD.

### Data collection

Data collection for the IDIs and the FGDs occurred between March and April 2018 and was preceded by two pilot FGDs done at one of the study facilities (Facility A). The pilot patients were excluded from the final study sample. Interview guides used for data collection from both HCWs and patients were informed by the literature [12,20] and pre-tested. The research team consisted of three trained fieldworkers: KH (male), BC and BB (females) in the presence of the researcher TM (a female medical doctor practicing public health in Botswana who is not fluent in isiXhosa). Field workers added additional probes as necessary. KH and BC managed the consent process. TM took field notes of non-verbal cues during the interviews to augment interpretation of the interview transcripts and enhance familiarity with the data. Patients and HCWs were not known to any member of the research team. Fieldworkers were non-practicing HCWs who had worked as field workers for at least 3 years and were able to practice the concept of bracketing and were sensitized to cultural norms when speaking to participants in this setting, especially older people. They were trained in T2D management, the study protocol, and data collection tools. Their qualifications as HCWs were not observed to influence participants away from talking freely. Rather the fact that the field workers lived locally, spoke local language made participants feel that they understood their challenges with self-management especially when it comes to managing diet and physical activity encouraged uninhibited contribution from participants. For example, when discussing unhealthy foods and using local herbs such as Aloe vera, participants were able to freely share their experiences. The lead researcher was present in the room during interviews and made field notes of interactions between fieldworkers and participants. The lead researcher also applied bracketing during data analysis and during interviews with HWCs.

### Data analysis

Data analysis was done using the Self-Management framework described by Lorig and Holman [12], based on work done by Corbin and Strauss [20] and guided by the seven-steps framework approach described by Gale et al. [21]. Recorded IDIs and FGDs were transcribed verbatim and translated into English by a fieldworker fluent in isiXhosa. Following familiarization, open coding of transcripts was done independently by two coders (TM) and (SO) using ATLAS.ti software version 7. SO (male) was not a member of the research team at the time of data collection. This initial inductive coding allowed for the unexpected; gave rise to memo creation; and gave researchers a holistic view of the data. Open codes developed from quotations were compared between the coders, agreed upon, and applied into an analytical framework informed by the three SM tasks of self-management. This was followed by applying framework codes to each transcript (closed coding) and data interpretation. The analysis was augmented with feedback from the supervisors (PD and TP) and field workers through debrief sessions.

### Ethical considerations

Ethics clearance was received from the University of the Western Cape biomedical ethics review board (BMREC Nb 130416-050). Local authorities granted permission to access the study sites (permit WC_2017RP50_730). Consent was administered to all participants. Confidentiality was ensured and maintained through anonymizing participant identifiers. In the FGDs, patients introduced themselves by their ‘clan’ names, which identify that one belongs to a certain grouping or family line in the community. For patients who wanted to use their real names, the information was removed during transcription and translation. Participants were treated with respect and continuously reminded that participation was voluntary, and that they could withdraw consent at any time. Those who did not consent were not coerced to participate, and their lack of consent did not in any way affect their access to healthcare. The interviews were conducted after the patients had received healthcare. No harm was done to participants, and they were made aware of where to go in case of concerns or complaints.

## Results

Findings are presented under five themes: i) behavioural/medical management; ii) role management; iii) emotional management; iv) barriers to self-management; and, v) solutions for enhancing self-management. Themes are summarized in Table 3 below:

**Table 3.** Summary of themes derived from the data.

Themes	Categories	Subcategories
Behavioural and medical (self-) management	Patient internal behavioural mediators	Lack of understanding, Low health literacy, Poor self-control
	External behavioural mediators	Marketing of unhealthy food, Cultural Influences, Affordability of healthy food
Role management	Interaction with health care professionals	Feeling disrespected by HCWs, Fear appeals, Dishonest behaviour
	Family support	Feeling unsupported
	Perceived stigma	Fear of disclosure, Self-isolation
Emotional management	Reactions to diagnosis	Shock/disbelief, Fear and depression
	Coping with diagnosis	Rationalization of diagnosis, Lack of acceptance, Seeking divine help, Non-adherence
Barriers to self-management	Patient-related mediators	Treatment side effects, Lack of education, Poor decision making, Entitlement mentality
	External factors	HCW attitude, Television advertising, Inadequate community services, Travel and migration, Staff shortage
Solutions for enhancing self-management	Patient-centred approaches	Individual consultations, Contextualised communication, Use of audio-visual material, Family support, Use available resources
	Community engagement	Community projects, Empowerment of street vendors, Patient advocacy
	Strengthened diabetes care services	Designated counselling services, Dedicated healthcare team, Treatment buddy, Community health worker, capacitation

### Behavioural and medical management

In this study, behavioural management is described in terms of patient-internal mediators (PIMs), external mediators (EMs), and disease control strategies. PIMs influenced how the patient engaged with the diagnosis, including lack of understanding of disease pathogenesis, lack of knowledge, and poor self-control. Poor self-control is illustrated by the quotes below from both a patient and HCW perspective:

I am part of a group who is just greedy. I was always going for the braai (barbecue) meat (P3:FGD1)

Ah patients are very stubborn as well when it comes to their diet (Int: giggles) they want to eat meat, they want to eat umphokoqo [starchy porridge] … with too much starch (P4 HCW female)

External mediators included the marketing of unhealthy foods, the cost of healthy food, and cultural influences. Some patients described feeling pressure to eat food considered not healthy when they attended cultural ceremonies such as funerals and weddings. Additionally, the belief that being overweight is a sign of good living had an influence on their eating habits. Marketing of unhealthy foods affected patients’ decision-making skills towards healthy food choices as perceived by healthcare workers, while patients had a common perception that healthy food was unaffordable. This in turn influenced patients’ ability to identify and use appropriate and available resources to support disease management through diet.

### Role management

This task requires patients to maintain or create new meaningful behaviours towards disease control, characterized by good relationships with their HCWs and good self-regulation [12]. Partnerships between patients and HCWs appeared negatively influenced by patients’ feeling disrespected by HCWs. This sometimes led to patients withholding important information, such as the use of herbal medicines and a lack of adherence to lifestyle modification from the HCWs.

I just drink a spoon of aloe extract and mix it with that metformin, and they find it at 8”, I just drink when I come to the clinic to fool the nurses … (P1 FGD2)

Then I always tell them you must be honest with me now, tell me exactly: have you followed it (lifestyle advice) … (P 2: HCW female)

Role management also requires HCWs to change their behaviours and attitudes and recognize the importance of the long-term partnership required for patients with long-term disease. To enhance patient adherence to their advice, some HCWs used fear appeals rather than motivational approaches during patient consultations:

But I always scare them that is my weapon, especially with diabetes I ask them … do you want to have just one leg? Do you want to be blind, do what is right so that you avoid these things so the scaring them to me works (P1 female HCW)

These did not always work for patients, however. Another important partnership influencing role management was family support, which significantly influenced how patients managed their disease. Some patients described poor family support, driven by a lack of understanding of T2D and its complications and criticism from some family members around dietary adherence:

They shout at me daily – dad you have not even tasted the food but want salt (P11 FGD4)

Forming strong partnerships was also influenced by perceived stigma and fear of being judged by others, such as intimate partners. This led to non-disclosure of the diagnosis and patient self-isolation from others, including family members:

And even at home, you are judged by your disease. So you see I stay alone and they always used to ask me, why would you stay alone when you are diabetic … because I do not want to be judged for my disease (P7 FGD3)

### Emotional management

This task recognizes the significant emotional impact of being diagnosed with a chronic disease on patients. Two categories were identified, including initial reactions to and coping with the diagnosis. Initial reactions to a diagnosis of T2D/HTN included those of shock, disbelief, and fear, which were expressed by participants across all FGDs:

I was shocked and I asked myself if the doctor tested me correctly, maybe he is making a mistake, I thought that he made a mistake … (P2 FGD1)

Various coping mechanisms were used, including finding explanations for why they would have the disease, e.g. hereditary, seeking divine guidance, not accepting the diagnosis, and non-adherence to medication. Sometimes patients failed to cope, leading to unresolved emotions for several years after diagnosis. These emotions were described, especially among the older population, and included stress and depression

The elderly people get scared and you will see that they are scared and stressed, they get down and depressed (P6 Female HCW)

The three themes described above demonstrate that patients diagnosed with T2D/HTN experience important challenges and barriers to self-management.

### Barriers to self-management

Two types of barriers were identified, including patient-related barriers and external barriers. The patient-related barriers included adverse treatment effects, lack of education reported by patients, poor decision-making, and entitlement mentality perceived by healthcare workers. This entitlement mentality seemed to affect patients’ abilities towards problem-solving and resource identification and utilization, as illustrated below:

My people have that belief in their minds that the government must do everything for them, they don’t want to do anything for themselves (P2 female HCW)

For example, it was thought by healthcare workers that patients spent some of their money on expensive commodities such as meat, alcohol, and cigarettes instead of healthy foods.

External barriers included HCW attitudes experienced by patients, television advertising, inadequate community services, travel and migration between service delivery points and patients’ residence, and staff shortages noted by healthcare workers. HCW’s attitude was a major concern for patients, who decried the disrespectful treatment by HCWs across the FGDs:

So do you understand I am here to get advice from her and then the way she responded to me it was as if I could just leave there and get smashed by a train … … (P8 FGD3)

In contrast, HCWs believed that telemarketing of unhealthy foods was a more important barrier to patients adopting a healthy lifestyle:

It is very difficult. What matters currently is commercials and what is seen on TV matters more than what we tell people in our institutions … (P2 female HCW)

Additionally, seeing patients at the facility and not being familiar with their home circumstances, travel or migration from the Eastern Cape to access treatment, and staff shortages were other important barriers noted by HCWs.

### Solutions to enhancing self-management

Three types of solutions for enhancing patients’ SM were found: patient-centred approaches, community engagement, and strengthened diabetes care services.

#### Patient-centred approaches

Patient-centred solutions were mainly suggested by HCWs. They included individually tailored consultations for struggling patients, contextualized communication, encouraging patients to use available and affordable local food resources for dietary requirements and family support. An example of contextualized communication was using an analogy that patients can understand e.g. the three-legged pot:

That pot must have those three legs in order for it to stand, if one leg breaks, it is no longer a pot … so we make that example into managing their hypertension (P1 HCW female)

Using movies as an audio-visual communication tool was suggested by HCWs who noted that even patients with low literacy watched movies and understood the storylines. They could therefore understand health messaging communicated using similar platforms.

You realize that wow, they actually understand what is happening in the movies … because mostly even people who are not educated are good at watching things (P6 female HCW)

This solution would allow HCWs to use this type of media to influence health behaviour through health marketing during TV programmes mostly watched by patients and their families. As families tend to watch television or movies together, this could also strengthen family support. Additionally, patients attending consultations accompanied by family members would also enhance family support and facilitate concurrent health education sessions.

#### Community engagement approaches

Patients and HCW suggested community-oriented and patient empowering SM enhancing solutions. Community engagement solutions included empowering street (food) vendors, engaging patients in community projects and advocacy, and capacitating community health workers (CHWs).

Street vendors sell mostly unhealthy convenience food, including hot dogs, braai meat, and fat cakes near health facilities. Empowering these street vendors towards selling healthy food to patients was considered an important solution:

It needs to be changed there first, and then also these little shops on the side, these Spaza shops (street vendors) and if we can go to these people also and see what they’re actually selling and giving them healthy alternatives (P2 female HCW)

The empowerment could be extended to patients through community projects such as youth-run vegetable gardens, to be self-sufficient in healthy food production and create employment:

I would like to agree with this lady, there is a lot of unemployed youth out on the street so if the government could develop a big project where each section has a garden planted by the youth for the elderly … … that would be very helpful. (P10 FGD3)

Patient-led advocacy through community campaigns to reduce the feeling of disempowerment was also suggested by patients:

Also, for campaigns to finally take place, for diabetics to finally stand up and even the ones in wheelchairs to push their chairs, and for us to not be under the table but on top of it. (P4 FGD2)

#### Strengthened diabetes care services

Under this category, suggested solutions included designated diabetes counselling services and a dedicated T2D healthcare team.

Measures to strengthen diabetes care such as designated counselling services like those provided for HIV patients were suggested by patients in this study.

You are there to test if you have HIV, so if they found that you are positive they first counsel you but with this thing, it just comes out … and it needs treatment immediately, do you understand? So where is the time for counselling? There is no time, right! (P11 FGD4)

A treatment buddy, similar to an HIV buddy was also suggested. These solutions would enable patients to cope with psychological emotions evoked by diagnosis of T2D/HTN and fear of their complications:

I think for people with fears of amputation should their diabetes go up, counselling would help like in the case of that lady so that her worries are not so dependent on that (P6 FGD1)

Types of counselling suggested included pre-test counselling, continuing counselling, and family counselling to enhance understanding of the implications of T2D diagnosis for patient self-management. Community healthcare teams dedicated to T2D/HTN consisting of a counsellor, a dietician, a health educator, a nutritionist, and nurses committed to providing quality care were recommended.

People from the club should have a team in the club like a dietician, counsellor, educator, nurses that love clubs … … (P6 female HCW)

## Discussion

In this study, we explored experiences, identified self-management barriers and solicited solutions for enhancing self-management of T2D/HTN from patients and their healthcare providers.

Behavioural/medical management was challenging for patients, mediated by factors internal and external to the patient. Poor patient self-control was the main internal challenge, driven by a lack of understanding and poor disease knowledge. This relationship between diabetes knowledge and poor self-management has been demonstrated in quantitative studies [22,23], including a positive association between diabetes education and self-management behaviours [24].

External influencers of behavioural/medical management included the marketing of unhealthy food and the perceived unaffordability of healthy food. Patients’ SM challenges were particularly around diet-related lifestyle modification. A US-based study among adults aged 18 years and above showed that advertising food through television strongly affects individual food choices [25]. This includes evidence from a study in Mexico which showed that consumption of advertised foods among mothers and their children is associated with frequency of broadcasted advertising, number of hours of watching television, and body mass index (BMI) [26]. However, evidence of the effect of advertising on adult T2D/HTN is lacking.

Role management also challenged patients, particularly the creation and maintenance of healthy behaviours and meaningful partnerships. Key contributors to these challenges were perceptions of disrespect by HCWs, lack of family support, and stigma. HCWs observed that patients remained non-adherent to medications and lifestyle advice. Different approaches were used to improve adherence, including the use of metaphoric and fear appeals, which are considered effective in positive behaviour change [27]. While HCWs believed that fear appeals enhanced role management, patients’ perceptions were contrary to this notion. Research suggests that fear appeals are effective when recipients have high self-efficacy but are ineffective or could even backlash when self-efficacy is low [28], even leading to maladaptive behaviours such as those shown in this study. A meta-analysis of qualitative and quantitative studies has shown that positive emotional appeals might prove more effective than cognitive appeals or negative emotional appeals [29], which supports experiences from our study participants.

Role management in this study was negatively affected by patients’ experiences of disrespectful HCW attitudes and poor communication. Feeling disrespected by healthcare providers was reported as a major reason for not honouring outpatients’ clinic appointments in a qualitative US study [30], which resonates with patients in this study. These challenges could be addressed by using a more patient-centred participatory and appreciative approach [31] that addresses the patient agenda, a priority neglected by HCWs but valued by patients [32].

Family support was considered important to role management by both patients and HCWs. The importance of family support in diabetes care and management is well captured in a commentary by Ahmed and Yeasmeen [33], who argue that ‘better health can be provided if the family is considered central to the management of diabetes’. Empirical evidence from South Africa has demonstrated a positive association between family support and following a diabetic meal plan, foot care, physical activity, and emotional management of T2D diagnosis, underscoring the importance of family support in T2D self-management [34].

Emotional management was a challenge for patients having to deal with the grief-reaction evoked by a T2D diagnosis, ranging from initial shock and denial to acceptance and coping with future disease complications. Grief reactions among T2D patients were described even three decades ago [35] and recently [36], highlighting the importance of acknowledging the emotional management of coping with the diagnosis.

Patients experienced several barriers due to internal and external factors. Patients and HCWs identified possible solutions to enhance SM. The SMART2D Self-Management framework [9] is considered a useful model for guiding the implementation of such solutions. The framework encapsulates four core SM tasks (medical, emotional, role, and lifestyle management); five SM skills (decision making, resource mobilization, taking action, problem-solving, and forming partnerships); and five mediators (perceived autonomy, perceived relatedness, self-efficacy, illness perception, and learning of self-management strategies). The framework embeds these requirements within the patient’s proximal environment. The barriers and solutions for optimal SM suggested in our study speak directly to all these factors.

## Study strengths and limitations

The study ensured trustworthiness as defined by Lincoln and Guba [37], encompassing transferability, credibility, dependability, and confirmability. The thick description of the study context, methods, and use of quotations from participants enhanced confirmability, while dependability is demonstrated through literature citations showing consistency with our findings. Credibility was shown through triangulation using data from patients and HCWs, collecting data at different times of the year, week and day to cater for the influence of seasonal migration, using two interviewers per recording session and different coders, which enhanced interpretation of the data. Field notes and personal reflections by the researcher enhanced reflexivity, reducing being judgemental.

While findings from this study are based on information provided by HCWs and patients in a specific locality and used a small number of participants, which may not reflect the views, experiences, and opinions of all HCWs and patients in this setting, the facilities where this study was conducted are among the largest provider of chronic disease management programmes for T2D/HTN patients in this area.

## Conclusion

In this study, both patients and providers found self-management to be complex in real life. Patients experienced challenges across three self-management tasks of behavioural and medical management, role management, and emotional management. Main challenges included lifestyle modification for disease self-management, poor partnerships with HCWs and family members, and post-diagnosis grief-reaction, cultural norms, poor HCW-patient communication, and disconnect between facility-based HCWs and patients’ lived experiences. Barriers to self-management included stigma, entitlement mentality, inadequate community services, and human resource shortages. Patients want empowering, predominantly community-based solutions to strengthen their self-management. Suggested solutions included dedicated diabetes/HTN services with multidisciplinary teams, counselling services, increased family support, patient advocacy, and contextualised communication using audio-visual technologies. A contextualized self-management framework that covers the essence of these solutions is recommended as an ideal multi-pronged approach to self-management in this setting.

## Policy recommendations

- Dedicated community-based multi-disciplinary healthcare team using well-trained CHWs
- Healthcare workers’ capacitation to use patient-centred consultation models such as motivational interviewing
- Provision of pre- and post-counselling services in the community for patients and families
- Introduction of a chronic disease buddy-system similar to the HIV buddy system to further strengthen patient self-management
- Chronic disease care programmes that feature patient-driven community projects and supports patient-led advocacy

## Paper context

Patients attending chronic disease clubs in South Africa are expected to practice self-management of their diseases, but they face significant challenges. We unravelled their experiences, challenges, and barriers to practising optimal self-management. They have previously not been asked to provide their voice on solutions to these. This study went a step further and solicited solutions to improve self-management from these patients and their providers experiencing self-management in one of the largest townships in South Africa.

## References

[1] Foreman KJ, Marquez N, Dolgert A, et al. Forecasting life expectancy, years of life lost, and all-cause and cause-specific mortality for 250 causes of death: reference and alternative scenarios for 2016-40 for 195 countries and territories. Lancet. 2018 Nov 10;392:2052–10. 30340847 10.1016/S0140-6736(18)31694-5 PMC6227505

[2] Jankovic J, Mirkovic M, Jovic-Vranes A, et al. Association between non-communicable disease multimorbidity and health care utilization in a middle-income country: population-based study. Public Health. 2018 Feb;155:35–42. 29306621 10.1016/j.puhe.2017.11.014

[3] World Health Organization (WHO). Diabetes [Internet] Geneva; 2021 [cited 2021 Jul 19]. Available from: https://www.who.int/news-room/fact-sheets/detail/diabetes

[4] Lin X, Xu Y, Pan X, et al. Global, regional, and national burden and trend of diabetes in 195 countries and territories: an analysis from 1990 to 2025. Sci Rep. 2020 Sep 8;10:14790. 32901098 10.1038/s41598-020-71908-9 PMC7478957

[5] Nojilana B, Bradshaw D, Pillay-van Wyk V, et al. Emerging trends in non-communicable disease mortality in South Africa, 1997–2010. S Afr Med J. 2016 Apr 1;106:58. 10.7196/SAMJ.2016.v106i5.10674 27138667

[6] Stokes A, Berry KM, Mchiza Z, et al. Prevalence and unmet need for diabetes care across the care continuum in a national sample of South African adults: evidence from the SANHANES-1, 2011–2012. PLoS One. 2017 Oct 2;12:e0184264. 28968435 10.1371/journal.pone.0184264 PMC5624573

[7] World Health Organization (WHO). Global status report on non-communicable diseases 2014 [Internet]. Geneva; 2014 Oct 26 [cited 2021 Jul 19]. p. 302. (Report No. WT 500). Available from: https://www.who.int/publications/i/item/9789241564854

[8] Stephani V, Opoku D, Beran D. Self-management of diabetes in Sub-Saharan Africa: a systematic review. BMC Public Health. 2018 Sep 29;18:1148 30268115 10.1186/s12889-018-6050-0 PMC6162903

[9] De Man J, Aweko J, Daivadanam M, et al. Diabetes self-management in three different income settings: cross-learning of barriers and opportunities. PLoS One. 2019 Mar 19;14:e0213530. 30889215 10.1371/journal.pone.0213530 PMC6424475

[10] Redman BK. Responsibility for control; ethics of patient preparation for self-management of chronic disease. Bioethics. 2007 Jun;21:243–250. 17845469 10.1111/j.1467-8519.2007.00550.x

[11] Banerjee M, Chakraborty S, Pal R. Diabetes self-management amid COVID-19 pandemic. Diabetes Metab Syndr. 2020 Jul–Aug;14:351–354. 32311652 10.1016/j.dsx.2020.04.013 PMC7194953

[12] Lorig KR, Holman H. Self-management education: history, definition, outcomes, and mechanisms. Ann Behav Med. 2003 Aug;26:1–7. 12867348 10.1207/S15324796ABM2601_01

[13] Hardman R, Begg S, Spelten E. What impact do chronic disease self-management support interventions have on health inequity gaps related to socioeconomic status: a systematic review. BMC Health Serv Res. 2020 Feb 27;20:150. 32106889 10.1186/s12913-020-5010-4 PMC7045733

[14] Aweko J, De Man J, Absetz P, et al. Patient and provider dilemmas of type 2 diabetes self-management: a qualitative study in socioeconomically disadvantaged communities in Stockholm. Int J Environ Res Public Health. 2018 Aug 22;15:1810. 10.3390/ijerph15091810 PMC6164476 30135373

[15] Guwatudde D, Absetz P, Delobelle P, et al.; SMART2D Consortium. Study protocol for the SMART2D adaptive implementation trial: a cluster randomised trial comparing facility-only care with integrated facility and community care to improve type 2 diabetes outcomes in Uganda, South Africa and Sweden. BMJ Open. 2018 Mar 17;8:e019981. 10.1136/bmjopen-2017-019981 PMC5879646 29550780

[16] Strategic Development Information and GIS Department. City of Cape Town – 2011 census suburb Khayelitsha [Internet]. Cape Town; 2013 Jul [cited 2021 Jul 19]. Available from: https://resource.capetown.gov.za/documentcentre/Documents/Maps%20and%20statistics/2011_Census_CT_Suburb_Khayelitsha_Profile.pdf

[17] Battersby J. The state of food insecurity in Cape Town. AFSUN Food Secur Ser. 2011 Sep 11;28;12.

[18] Werfalli M, Murphy K, Kalula S, et al. Current policies and practices for the provision of diabetes care and self-management support programmes for older South Africans. Afr J Prim Health Care Fam Med. 2019 Aug 29;11:e1–e12 10.4102/phcfm.v11i1.2053 PMC6739530 31478747

[19] Creswell JW. Qualitative inquiry and research design: choosing among five approaches. 3rd ed. Washington (DC): Sage; 2013.

[20] Corbin JM, Strauss A. Unending work and care: managing chronic illness at home. Contemp Soc. 1989 Nov 1;18:945.

[21] Gale NK, Heath G, Cameron E, et al. Using the framework method for the analysis of qualitative data in multi-disciplinary health research. BMC Med Res Methodol. 2013 Sep 18;13:117 24047204 10.1186/1471-2288-13-117 PMC3848812

[22] Bruce DG, Davis WA, Cull CA, et al. Diabetes education and knowledge in patients with type 2 diabetes from the community: the fremantle diabetes study. J Diabetes Complications. 2003 Mar–Apr;17:82–89. 12614974 10.1016/s1056-8727(02)00191-5

[23] Kueh YC, Morris T, Borkoles E, et al. Modelling of diabetes knowledge, attitudes, self-management, and quality of life: a cross-sectional study with an Australian sample. Health Qual Life Outcomes. 2015 Aug 19;13:129 26286395 10.1186/s12955-015-0303-8 PMC4543474

[24] Juarez LD, Presley CA, Howell CR, et al. The mediating role of self-efficacy in the association between diabetes education and support and self-care management. Health Educ Behav. 2021 Apr 24; DOI:10.1177/10901981211008819 PMC8542053 33896236

[25] Zimmerman FJ, Shimoga SV. The effects of food advertising and cognitive load on food choices. BMC Public Health. 2014 Apr 10;14:342 24721289 10.1186/1471-2458-14-342 PMC4021209

[26] Bacardí-Gascón M, Díaz-Ramírez G, Cruz López B, et al. TV food advertisements’ effect on food consumption and adiposity among women and children in Mexico. Nutr Hosp. 2013 Nov 1;28:1900–1904 24506366

[27] Phillips BJ, Mcquarrie EF. Impact of advertising metaphor on consumer belief: delineating the contribution of comparison versus deviation factors. J Advert. 2009 Apr 1;38:49–62.

[28] Peters GY, Ruiter RAC, Ten Hoor GA, et al. Towards consensus on fear appeals: a rejoinder to the commentaries on Kok, Peters, Kessels, ten Hoor, and Ruiter (2018). Health Psychol Rev. 2018 Jun 5; 12:151–156 29558243 10.1080/17437199.2018.1454846

[29] Hornik J, Ofir C, Rachamim M. Quantitative evaluation of persuasive appeals using comparative meta-analysis. Commun Rev. 2016 Jul 11;19:192–222.

[30] Lacy NL, Paulman A, Reuter MD, et al. Why we don’t come: patient perceptions on no-shows. Ann Fam Med. 2004 Nov–Dec;2:541–545. 15576538 10.1370/afm.123 PMC1466756

[31] Ghaye T, Melander-Wikman A, Kisare M, et al. Participatory and appreciative action and reflection (PAAR) - democratizing reflective practices’. Reflective Pract. 2008 Nov 19;9:361–397.

[32] Macdonald L, Stubbe M, Tester R, et al. Nurse-patient communication in primary care diabetes management: an exploratory study. BMC Nurs. 2013 Sep 13;12:20. 24028348 10.1186/1472-6955-12-20 PMC3856446

[33] Ahmed Z, Yeasmeen F. Active family participation in diabetes self-care: a commentary. Diabetes Manage. 2016 Oct;6:104–107.

[34] Werfalli MM, Kalula SZ, Manning K, et al. Does social support effect knowledge and diabetes self-management practices in older persons with Type 2 diabetes attending primary care clinics in Cape Town, South Africa? PLoS One. 2020 Mar 13; 15:e0230173 32168342 10.1371/journal.pone.0230173 PMC7069645

[35] Brown SA. Diabetes and grief. Diabetes Educ. 1985;11:53–57. Summer. 3852733 10.1177/014572178501100211

[36] Ferrara L, Singleton J, Yang K, et al., Grieving the loss of self: challenges in type 2 diabetes mellitus self-management. J Dr Nurs Pract. 2018;11:25–34. 32745041 10.1891/2380-9418.11.1.25

[37] Lincoln E, Guba YS. Naturalistic inquiry. Newbury Park (CA): SAGE Publications Inc; 1985.

